# Is vertebral rotation correction maintained after thoracoscopic anterior scoliosis surgery? A low-dose computed tomography study

**DOI:** 10.1186/s13013-017-0131-1

**Published:** 2017-08-17

**Authors:** Luke A. Reynolds, Maree T. Izatt, Eric M. Huang, Robert D. Labrom, Geoffrey N. Askin, Clayton J. Adam, Mark J. Pearcy

**Affiliations:** Paediatric Spine Research Group, Institute of Health and Biomedical Innovation at Centre for Children’s Health Research, Queensland University of Technology and Mater Health Services, Level 5, 62 Graham Street, South Brisbane, 4101 Queensland Australia

**Keywords:** Intravertebral rotation, Intervertebral rotation, Rotation correction, Computed tomography, Adolescent idiopathic scoliosis, Thoracoscopic anterior spinal fusion, TASF

## Abstract

**Background:**

Axial vertebral rotation is a key characteristic of adolescent idiopathic scoliosis (AIS), and its reduction is one of the goals of corrective surgery. Recurrence of deformity after surgical correction may relate to rotation changes that occur in the anterior vertebral column after surgery, but whether any change occurs within the fused segment or in adjacent unfused levels following thoracoscopic anterior spinal fusion (TASF) is unknown. An analysis of measurements from an existing postoperative CT dataset was performed to investigate the occurrence of inter- and intra-vertebral rotation changes after TASF within and adjacent to the fused spinal segment and look for any relationships with the Cobb angle and rib hump in the two years after surgery.

**Methods:**

39 Lenke Type 1 main thoracic patients underwent TASF for progressive AIS and low dose computed tomography scanning of the instrumented levels of the spine at 6 and 24 months after surgery. Vertebral rotation was measured at the superior and inferior endplates on true axial images for all vertebral levels in the fused segment plus one adjacent level cranially and caudally. Intra-observer variability for rotation measurements was assessed using 95% limits of agreement to detect significant changes in inter/intra-vertebral rotation.

**Results:**

Significant local changes in inter- and intra-vertebral rotation were found to have occurred between 6 and 24 months after anterior surgical fusion within the fused spinal segment, albeit with no consistent pattern of location or direction within the instrumented fusion construct. No significant en-bloc movement of the entire fused spinal segment relative to the adjacent un-instrumented cranial and caudal intervertebral levels was found. No clear correlation was found between any vertebral rotation changes and Cobb angle or rib hump measures.

**Conclusions:**

Localised inter- and intra-vertebral rotation occurs between 6 and 24 months after TASF, both within the instrumented spinal segments and in the adjacent un-instrumented levels of the adolescent spine. The lack of measurable en-bloc movement of the fused segment relative to the adjacent un-instrumented levels suggests that overall stability of the instrumented construct is achieved, however the vertebrae within the fusion mass continue to adapt and remodel, resulting in ongoing local anatomical and biomechanical changes in the adolescent spine.

## Background

Adolescent idiopathic scoliosis (AIS) is a complex three-dimensional deformity that appears in early adolescence and can progress most rapidly during periods of growth. Various techniques have been developed for the treatment of progressive scoliosis including bracing, casting, exercise prescription and surgical correction employing posterior and/or anterior approaches to the spine. Scoliosis correction surgery aims to obtain a solid fusion mass across the instrumented spinal segments to maintain the correction achieved intra-operatively and prevent further deformity progression post-surgery. Axial vertebral rotation combined with rib cage deformity are major contributors to the trunk deformity found in AIS patients, and computed tomography (CT) has been found to be superior to radiographs for the accurate quantification of this parameter [1–3]. In addition to torsion within the spinal joints, rotary distortion is also found within the individual vertebrae (intravertebral rotation) which combine to contribute to the overall axial rotation of the apical regions of the spinal deformities in those with structural scoliosis [3, 4]. Clinically, the resulting trunk asymmetry is monitored non-invasively by measurement of the rib hump. Defining a consistent relationship between vertebral rotation and the clinically measured rib hump has proven difficult to date, with reductions in vertebral rotation measures after surgery not associated with similar levels of improvement in the rib hump [5–8].

Since the mid 90s, anterior thoracoscopic techniques have evolved as an alternative to posterior instrumentation for the correction of select types of scoliotic curves [4, 9–13]. Advantages of the thoracoscopic anterior spinal fusion (TASF) technique include decreased blood loss and implant density, less scarring, shorter hospital stay, restoration of the sagittal profile, reduced chest wall morbidity and maintenance of respiratory function [11–20]. The TASF method of correction for progressive Lenke type 1 scoliosis involves a multi-level discectomy, instrumentation using a single titanium rod (placed antero-laterally along the convexity of the scoliotic major curve) and vertebral body screws, and the insertion of bone graft to promote interbody fusion after surgery. Rib humps measured before surgery for the patient group suitable for TASF have been reported in the range of mean 14.6°–16.5° and are corrected to mean 6.4°–9.0° after surgery [18, 21–23].

A prior intervertebral (IV) fusion study [24] on 43 TASF patients from the same consecutive patient series, using a single low-dose CT scan at 24 months after surgery, found that in this patient group the fusion mass was not necessarily large, nor filled the entire inter-vertebral disc space. Despite this, a satisfactory fusion was found to have been achieved in this surgical group which was the impetus for the more extensive IV fusion study with multiple time points which has provided the CT dataset for the current study.

Loss of deformity correction including rib hump recurrence may be observed after anterior or posterior instrumented scoliosis correction surgery [18, 25–27], but the mechanism of any recurrence is unclear. Pratt et al. [25] reported that half of the rib hump correction achieved after posterior scoliosis surgery (27 patients) was lost by 1 year after surgery. Cui et al. [26] used CT to evaluate any loss of 3D correction after posterior pedicle screw surgery (27 patients) and found a mean 2.7° loss of apical vertebral rotation. Samdani et al. [27] identified a 5° or greater recurrence of rib hump after posterior scoliosis correction in 16 of 103 cases and attributed it to a combination of changes in rib morphology and also a loss of correction within the fused levels during the 2 years after surgery. Hay et al. [18] analysed 106 patients after TASF and reported a mean 1.4° loss of rib hump correction after surgery; however, there were 10% of the 106 cases identified with a 5° or greater rib hump recurrence, with the largest increase reported as 12°. Despite some suggested explanations as to the mechanisms and source of any loss of trunk asymmetry correction after fusion surgery, no prior studies have analysed the intra- and inter-vertebral rotation of the vertebrae included in the instrumented segment of the spine during the 24 months following scoliosis fusion surgery. The purpose of this study was to use the superior modality of CT in an available dataset to investigate, in patients who have undergone anterior selective thoracic fusion, whether (i) any loss of rotational or Cobb angle correction occurred in the 6 to 24 months post-operative period, either within the instrumented fusion construct or at the adjacent un-instrumented levels above and below the construct and (ii) were any inter/intra-vertebral rotation changes correlated with clinical ribcage rotation measurements (rib hump).

## Methods

### Study cohort

A single centre study of consecutive patients undergoing TASF surgery for progressive AIS is being undertaken for the primary purpose of evaluating inter-body fusion. Ethics approval was granted for 3 years, to enroll consecutive patients to have low-dose protocol CT scans of the instrumented thoracic spine levels plus 1 vertebral level above and below the construct at 6, 12 and 24 months after surgery. The current study is an adjunct project using only the 6 and 24 months postoperative scans from the main study dataset. During the 3 years recruitment phase of the study, all 49 (42 female, 5 male) patients who had TASF surgery at our centre were invited to participate. Inclusion criteria of [1] progressive adolescent idiopathic scoliosis diagnosis, [2] Lenke type 1 main thoracic Cobb angle > 45°, and [3] ability to attend our centre for all review appointments until minimum 2 years after surgery. Of the 49 invited, 40 (81.6%) agreed to participate and met the inclusion criteria.

### Surgical approach

**Fig. 1:**
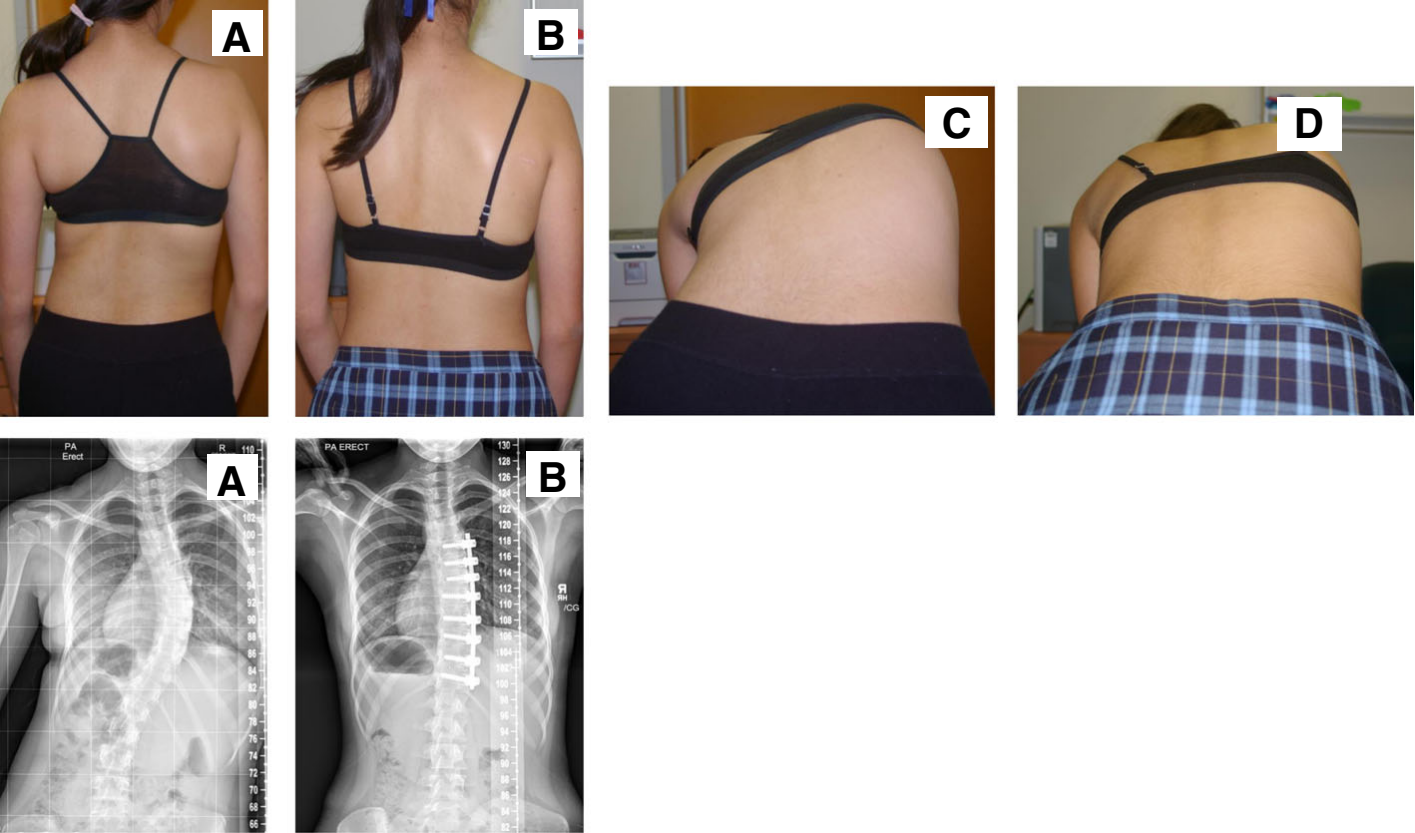
Photographs and radiographs of a typical TASF case (**a**) standing before and (**b**) standing after surgery, (**c**) forward bending before and (**d**) forward bending after surgery

The scoliosis correction surgeries were performed by authors GNA and RDL at Mater Health Services, Brisbane, Australia. Thoracoscopic anterior instrumented fusion surgery is based on the technique first described by Picetti et al. [11, 12] and aspects of its use at our centre have been reported previously [16–20, 24]. All patients in the study had a single 5.5 mm pure titanium rod and vertebral body screws implanted thoracoscopically (Legacy, Medtronic Sofamor Danek, Memphis, TN) and insertion of milled allograft bone following disc clearance to achieve curve correction using a standard compression technique. The instrumented vertebral levels were chosen to include the end vertebrae of the major scoliotic curve only, leaving any adjacent compensatory curves free to adjust postoperatively. Radiographs and photographs of a typical TASF case before and after surgery are shown in Fig. 1.

### Post-operative imaging

The CT scans used in the current study were performed at 6 and 24 months post-operatively for each patient. To ensure participants in the study did not receive a higher overall ionising radiation dose during the 24-month follow-up period than those receiving standard care, a minimal radiographic protocol was employed for study participants. This ensured the frequency of plain radiographs after surgery was reduced from standard practice (8–10 radiographs during the 2-year post-operative period), to 2 radiographs only (1 single standing postero-anterior (PA) radiograph prior to discharge from hospital after surgery and a single PA radiograph 6 months after surgery). The low-dose CT scans were performed with the patient supine with the imaging window spanning the instrumented levels and the superior and inferior immediately adjacent vertebrae only. The CT scanning parameters were identical for all post-op CT scan time points; Voltage 80–120 KVp, current 50–65 mA, slice thickness 1.00–1.25 mm with 2 scanners used during the data collection period of the study (64-slice Philips Brilliance and 64-slice GE Lightspeed VCT). The scanning protocols were set to ensure the total effective dose for each patient did not exceed 1.0 mSv during the 2-year duration of the study. This was set to comply with the ARPANSA code of practice which states that a child research participant’s effective radiation dose must not exceed 0.5 mSv in a year. Total effective dose takes into consideration all the tissues and organs in the field being imaged and their individual sensitivities to the incoming radiation. As part of everyday living, humans are exposed to naturally occurring background radiation and in Queensland, Australia this is reported to be 2.0 mSv annually. The commissioned dose report required for ethical approvals of the main study therefore concluded that the risk to participants was very low.

### Pre- and post-operative rib hump and Cobb angle measurements

The rib hump was measured using a simple Scoliometer [28–30] or smartphone [31] with the patient in the forward bending position before surgery and at 6 and 24 months after surgery. Cobb angles were measured on the available standing PA radiographs (before surgery and 6 months after surgery) and on reformatted coronal plane images produced from the 6 to 24 months post-surgery CT scan data using a method that has been described previously [19, 24, 32, 33]. Whilst it is known that the Cobb angle is reduced in supine compared to standing [34, 35], a valid comparison of changes between 6 and 24 months could be made using the supine CT data at these time points during the post-operative period.

### Image analysis

**Fig. 2:**
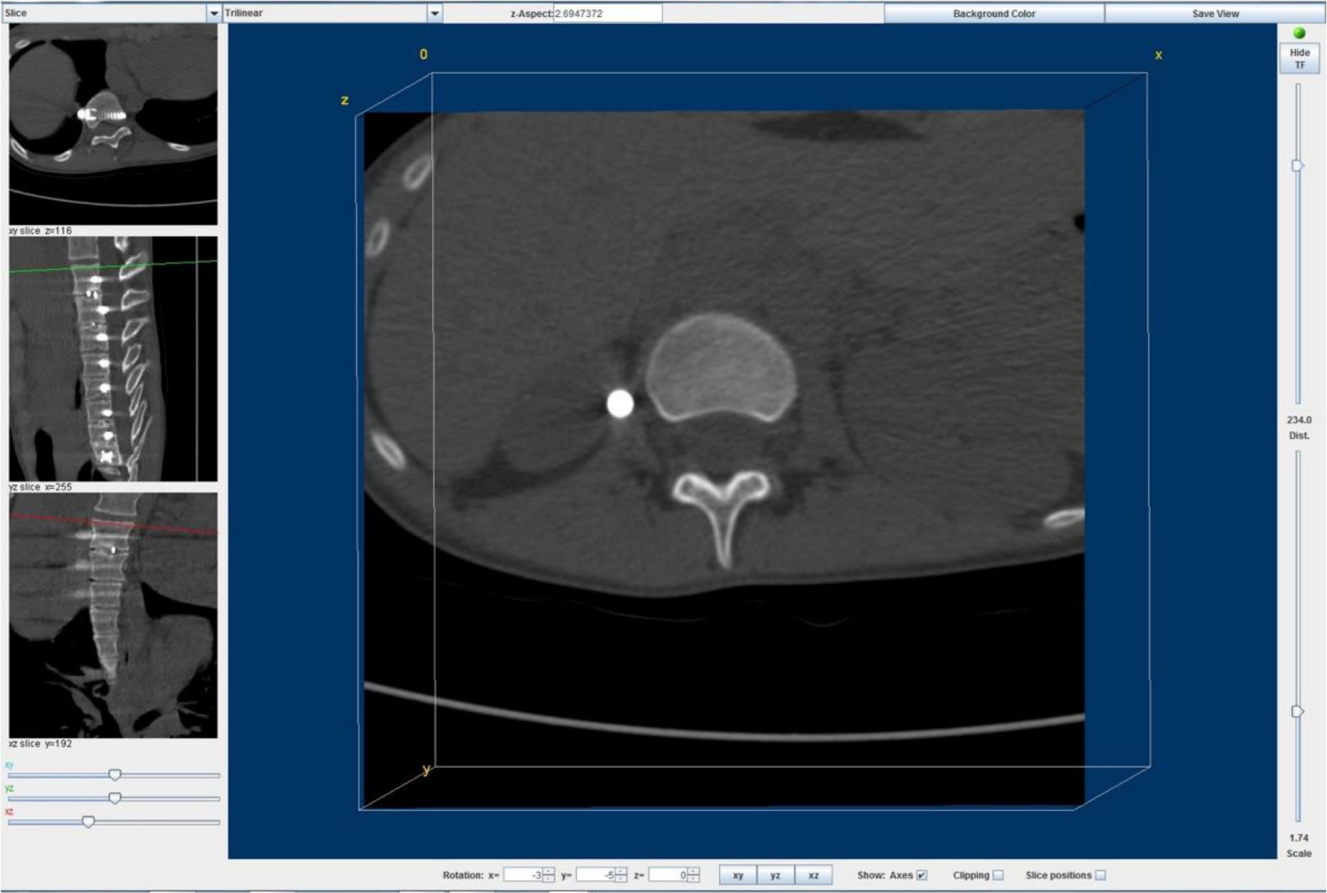
An example of a true axial image of the inferior endplate of L1. The large, true axial, image is obtained by modifying the plane of viewing in the frontal and sagittal planes with respect to the smaller images to the left. Rotation in the transverse plane was analysed in relation to the arbitrary CT scanner bed plane, thus allowing the measurement of relative inter-endplate rotations within patients

Using three-dimensional multi-planar reconstruction software (ImageJ 1.50b, National Institutes of Health, USA), ‘true axial’ (i.e. with the image orientation adjusted to lie in the plane of the endplate) images of all endplates within the fused spinal levels and in the adjacent un-instrumented levels were generated by a single observer for both the 6 and 24 months post-operative CT scans (Fig. 2).

**Fig. 3:**
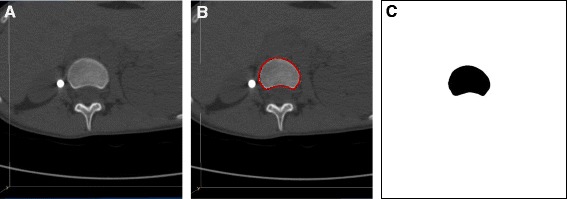
True axial images of vertebral endplates were segmented using the ImageJ software to produce binary images that could be processed by the rotation symmetry algorithm. **a.** True axial image. **b.** True axial image with polygonal outline of vertebral endplate. **c.** Binary image of vertebral endplate. The *bright white circle* in images A and B is a transverse section of the titanium rod

**Fig. 4:**
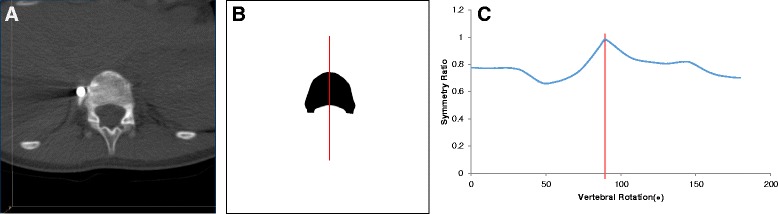
Analysis using the symmetry ratio algorithm shows that the superior endplate of the T12 vertebral body (**a**, **b**) is aligned at 89.8° (*vertical red line*) from the frontal plane (**c**), by convention for this study, the CT Scanner Bed

Using the true axial images, all endplate profiles were then manually traced using the ImageJ Polygonal Line tool (Fig. 3) and then each endplate’s axial rotation was measured using a custom written rotation symmetry plugin for the same software (Fig. 4). The rotation symmetry algorithm for the ImageJ program [36] is based on the premise that the axis of maximum symmetry of a vertebral cross-section defines the angular orientation of the vertebra and allows fully automated measurement of rotation for closed vertebral cross-sections, thus avoiding any observer error associated with the rotation measurement itself.

**Fig. 5:**
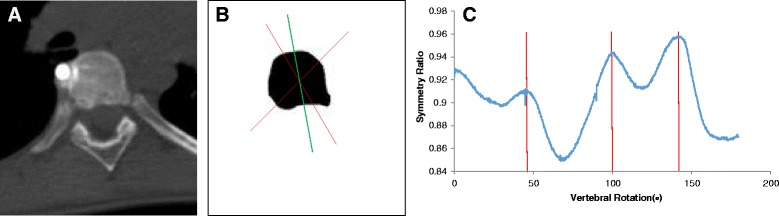
An example of anatomically impossible values of vertebral rotation given by the rotation symmetry program. This irregularly shaped outline (**b**) of a vertebral endplate (**a**) is more likely rotated at 100.3° than 45.4° or 142.1° (**c** *vertical red lines*)

On some occasions, the symmetry algorithm used to measure endplate rotations would give ambiguous rotation angles because there was more than one possible axis of near-symmetry through a particular endplate outline. An example of this is shown in Fig. 5. In these cases, manual inspection of the symmetry ratio versus rotation angle graph (which is an output of the algorithm) was used to choose the correct rotation angle (Fig. 5).

Where pedicles were visible in the true axial images of end plates (i.e. the superior vertebral endplates), the manual polygonal outline was drawn to include and cross the pedicle at its narrowest point (Fig. 5).

To analyse intra- and inter-vertebral rotation, each vertebral endplate rotation was measured relative to the anteroposterior reference line perpendicular to the CT scanner bed. When comparing the rotations between pairs of adjacent endplates, inter-vertebral rotations are equivalent to intra-discal rotations, and comprise the relative rotation between the inferior endplate of the cephalad vertebra and the superior endplate of the adjacent caudal vertebra. Intra-vertebral rotations are rotational deformities occurring within an individual vertebra, between its superior and inferior end-plates.

**Fig. 6:**
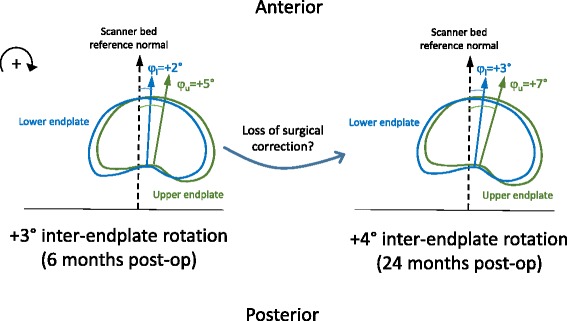
Sign convention for measurement of inter-endplate rotations; *clockwise* endplate rotations are positive viewed from above. Endplate rotations are measured relative to the scanner bed reference normal. Inter-endplate rotation = φu-φl where φu and φl denote *upper* and *lower* endplate rotations, respectively. In the example given, inter-endplate rotation at a vertebra/disc level below the *curve apex increases* from +3° at 6 months post-surgery to +4° at 24 months post-surgery, representing a small (non-significant in this example) loss of correction over time at the level in question

Viewed from above at a single time point, clockwise rotation of a cephalad endplate relative to its adjacent caudal endplate is positive inter-endplate rotation, and anti-clockwise rotation of a cephalad endplate relative to its adjacent caudal endplate is negative inter-endplate rotation. Thus, in a right thoracic scoliotic major curve, inter-endplate (intra-vertebral/intra-discal) rotations tend to be negative above the curve apex and positive below the curve apex. From this convention, it was noted that increases in the magnitude of inter- or intra-vertebral rotation between 6 and 24 months post-surgery (i.e. negative changes above the apex and positive changes below the apex) indicate a loss of surgical correction towards pre-operative deformity. The sign convention and an illustrative example of changes in inter-endplate rotation over time are given in Fig. 6.

If the appropriate rotation angle could not be identified by manually inspecting the endplate outline, the patient was excluded from further analysis. The values of endplate rotation from the remaining datasets were then analysed to calculate changes in inter- and intra-vertebral rotation between 6 and 24 months after surgery.

### Statistical analysis

Even though the rotation symmetry algorithm used to measure endplate rotations is fully automated and therefore is not subject to observer measurement error, measurement variability is still introduced by the manual generation of the true axial images in the plane of each vertebral endplate, and by the manual tracing of endplate profiles. Intra-observer variability for inter-endplate rotation change between 6 and 24 months post-surgery was therefore assessed using the 95% limits of agreement method described by Bland and Altman [37, 38]. Intra-observer variability was assessed by analysing the absolute difference in post-surgical rotation changes, across the 6 and 24 months post-surgical time-points, within individual patient CT datasets. The same observer measured the changes in inter-endplate rotation at corresponding levels between 6 and 24 months post-surgery in 5 patients; then re-measured the changes in inter-endplate rotation in those same patient CT datasets after an interval of at least 2 weeks. Thus, intra-observer variability was calculated as

$$ \varDelta \alpha = \left|{\alpha}_n - {\alpha}_m\right| $$

where *n* and *m* are successive inter-endplate rotation change values obtained by the same observer using the full method above. The 95% confidence intervals for intra-observer variability were calculated as 1.96 × SD_intra_ where SD_intra_ is the standard deviation of the intra-observer differences *Δα*.

## Results

One participant failed to attend appointments after surgery, such that a total of 39 patients (35 female, 4 male) had data available for the current analysis. Of the 39, 3 were excluded due to incorrect CT slice width protocols, 1 failed to have the scan at 24 months and 1 was excluded due to poor image quality. Two additional patient datasets were subsequently excluded due to endplate rotation values that could not be measured with the rotation symmetry program due to ovoid and misshapen vertebral bodies, leaving 32 patients for analysis (29 female, 3 male) in the current study.

The mean age at surgery was 15.5 years, SD 1.8 (range 12.5–19.2). All 32 patients had right thoracic major curves classified as Lenke 1A for 21 patients, 1B for 7 patients and 1C for 4 patients. The mean number of levels instrumented was 7.6, SD 0.8 (range 6–9). The proximal extent of the rod was to T4 (*n* = 2), T5 (*n* = 14), T6 (*n* = 14) or T7 (*n* = 2). The distal instrumented level was to T11 (*n* = 5), T12 (*n* = 21) or L1 (*n* = 6). No bracing was used after surgery which is standard practice after thoracoscopic anterior surgery at our centre. No mechanical complications (rod fractures or screw pull-outs) occurred for any of the participants in the study.

### Changes in rib hump and Cobb angle measurements

The mean rib hump before surgery was 15.0°, SD 3.5 (range 8–22) and was corrected to mean 6.8°, SD 2.4 (range 3–13) at 6 months and mean 8.3°, SD 2.6 (range 4–15) at 24 months after surgery. The mean change in the clinically measured rib hump between 6 and 24 months post-surgery was 1.6°, SD 1.3 (range 0–5°), similar to a previous published larger TASF cohort [18], with 7 patients in the current study recording a rib hump change of 3° or greater.

The main thoracic Cobb angle before surgery was mean 55.8°, SD 5.9 (range 43–69), which was corrected to mean 21.0°, SD 7.1 (range 8–35) after surgery, as measured on the available standing PA radiograph taken 6 months after surgery. The change in Cobb angle measured from the reformatted supine CT data between 6 and 24 months post-surgery ranged from −6 to 3°. The patient that showed a −6° change in Cobb angle was the only patient to show a change greater in magnitude than 3° and, furthermore, had no statistically significant inter-endplate rotations within or adjacent to the instrumented spinal levels.

### Intra-observer variability

Intra-observer variability for change in inter-endplate rotation between 6 and 24 months post-surgery was ±4.2° with a 95% confidence interval of ±8.2°. This 95% confidence interval of ±8.2° was used to define measurable changes in vertebral rotation between 6 and 24 months post-surgery. Only values greater in magnitude than an 8.2° change were deemed significant, as values of lesser magnitude could not (with 95% confidence interval) be considered reliable measures of actual change.

### CT image analysis: inter- and intra-vertebral rotations

**Fig. 7:**
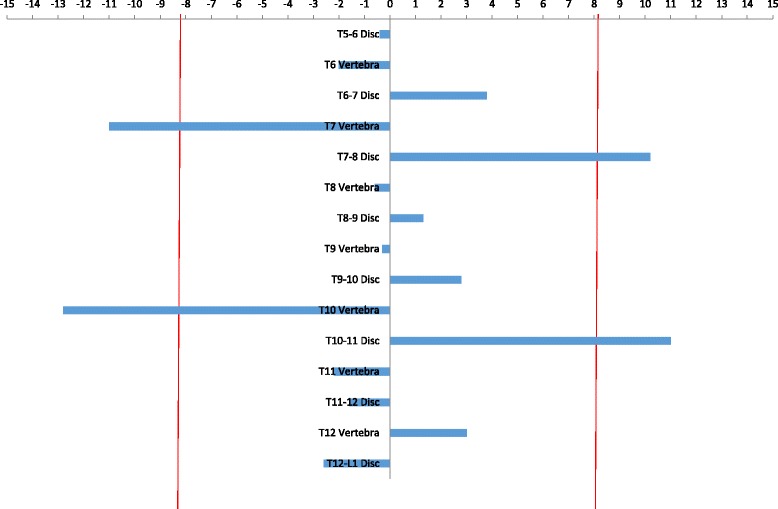
Four statistically significant changes of inter- or intra-vertebral rotation were measured in this individual patient between the 6- and 24-month post-operative low-dose CT scans following T6-T12 TASF with apex T9. (the *red vertical lines* indicate 95% confidence interval, and four values were larger than this.)

**Fig. 8:**
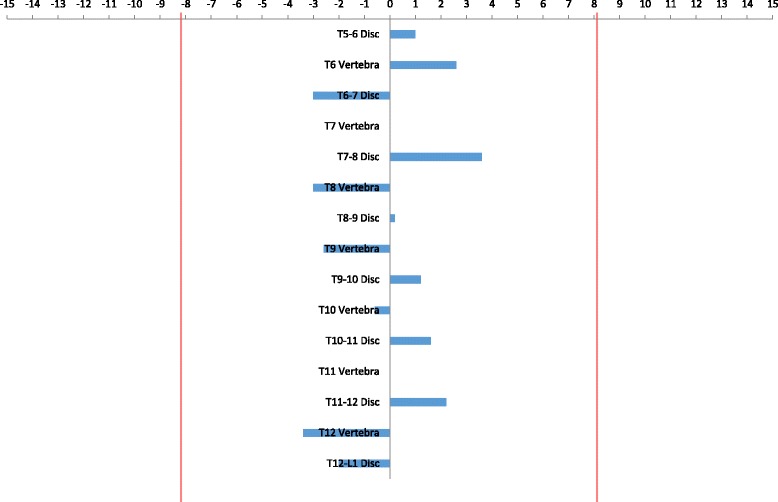
No statistically significant changes in inter- or intra-vertebral rotation were measured in this individual patient between the 6- and 24 month post-operative low-dose CT scans following T6-T12 TASF with apex T8-T9. (the *red vertical lines* indicate 95% confidence interval, and no values were larger than this.)

Of the 32 participants analysed, there was 1 patient with a measurable change in inter-vertebral rotation in the first un-instrumented disc above the construct using the 95% confidence interval of ±8.2°. Only one statistically significant change in intra-vertebral rotation was measured in the first un-instrumented vertebra below the construct between the 6- and 24-month post-operative scans, whilst there were no significant changes in rotation for the first un-instrumented disc inferior to the construct across the patient datasets (Table 1). Examples of patients with and without statistically significant inter- or intra-vertebral rotation changes between the 6- and 24-month post-operative low-dose CT scans are given in Figs. 7 and 8.

**Table 1:**
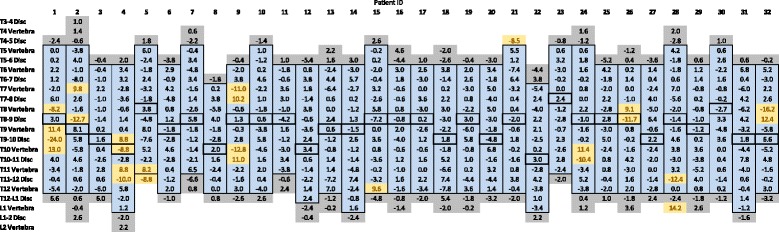
The inter- and intra-vertebral rotation changes for all 32 patients, between 6 and 24 months post-surgery by vertebral level. Statistically significant (>95% limit of intra-observer agreement) changes in inter- and intra-vertebral rotation between 6 and 24 months after surgery are coloured *yellow*. The *blue squares* outlined in bold represent the instrumented spinal levels (be they inter- or intra-vertebral levels), and *grey squares* are the un-instrumented levels caudal or cephalad to the instrumented fusion construct. When examined with respect to the apical vertebra location (level marked with bold borders for each patient), positive values below the apex and negative values above the apex both indicate increases in the inter/intra-vertebral rotation between 6 and 24 months post-surgery (i.e. loss of surgical correction)

Using the ±8.2° value as a cut-off for significance, 11 of the 32 patient datasets showed at least 1 measurable change in inter- or intra-vertebral rotation between 6 and 24 months post-operatively. Amongst the patient datasets, several large changes in inter- and intra-vertebral rotation were grouped around two or three vertebral levels with changes in opposite directions and of approximately equal magnitude.

The mean changes in intra-vertebral rotation between 6 and 24 months post-surgery for the superior and inferior vertebrae adjacent to the fused construct were 1.3° (range −5.4° to 6.8°) and −0.8° (range −7.8° to 8.2°), respectively. The mean changes in inter-vertebral rotation between 6 and 24 months post-surgery for the adjacent un-instrumented discs superior and inferior to the fused spinal levels were −1.0° (range −8.5° to 3.8°) and −0.5° (range −8.8° to 6.6°), respectively. The mean change across all patients between 6 and 24 months post-surgery at the apex of the curve, be it discal or vertebral, was 0.2° (range −11.7° to 8.1°). Therefore, there was no clear pattern of en-bloc movement of the instrumented levels relative to the adjacent un-instrumented spine, with insignificant mean changes in inter- and intra-vertebral rotations at the superior and inferior junctions of the fused and un-fused spine between 6 and 24 months after surgery (Table 1).

Manual inspection of the data showed no clear links between any of the significant 6–24-month inter- and intra-vertebral rotation changes and either the Cobb angle or rib hump measurements.

## Discussion

Correction surgery for progressive scoliotic deformity aims to halt progression, reduce the magnitude of the deformity in three dimensions and improve trunk appearance. The rotation of the vertebral column in structural scoliosis contributes to the trunk deformity in these patients. It is not known, however, if post-operative rotational change within or adjacent to the fused spinal levels contributes to recurrence of trunk deformity in the years following instrumented surgical fusion.

The few published CT studies on recurrence of deformity after surgical fusion procedures for scoliosis have suggested mechanisms and sources for the recurrence of trunk and/or vertebral deformity. Xiong et al. [39] in an early CT study of the apical vertebra on a small patient sample (*n* = 10) suggested that remodelling of the scoliotic vertebral body after Cotrel-Debousset instrumentation contributed to the loss of the correction that was being reported in the first few years after surgery by prior studies [40, 41]. Cui et al. [26] examined the loss of three-dimensional correction after posterior pedicle screw surgery using the Aaro and Dahlborn method [2] of vertebral rotation measurement on reconstructed axial CT images for 27 patients. In common with the current study, Cui et al. used reconstructed CT images to measure vertebral rotation in the plane of the endplate. However, only the rotation of the upper instrumented vertebra and the apical vertebra were measured to generate a relative apical vertebral axial rotation value as the difference between them. The current study used a measurement tool that primarily considered anterior anatomical structures [36], as opposed to the Aaro and Dahlborn method used by Cui et al., which includes posterior, potentially deformed [42], anatomical structures. Furthermore, the current paper uniquely measured the endplate rotation of all instrumented end-plates along with the adjacent superior and inferior un-instrumented end-plates so as to generate a complete set of inter- and intra-vertebral rotation changes for each patient during the 2 years following surgery. Our approach allowed an analysis of both the mechanisms and sources of deformity recurrence which has not been achieved in prior studies. As such, we have been able to localise where rotational change may be occurring or recurring within or adjacent to the instrumented spine.

Statistically significant changes in inter- or intra-vertebral rotation during the 2 years after surgery were found to be sporadic in relation to vertebral levels, the apex of the corrected curve or the cephalad and caudal limits of the fused spinal levels (Table 1). These statistically significant changes to rotation within both vertebrae and discs were identified as greater than the 95% confidence interval of ±8.2° (Fig. 6). Although stringent, using the 95% confidence interval of ±8.2° to define significant rotational changes adds rigour to the findings, given the potential for said findings to influence surgical management in the future. If one standard deviation had been used as opposed to two, which equates to a confidence interval of 68% instead of 95%, the number of significant values for changes to inter- and intra-vertebral rotation between 6 and 24 months post-surgery, across the 32 patients analysed, would have been higher. However, again no pattern of rotational changes in relation to the apex of the corrected curves or the cephalad and caudal limits of the fused vertebral levels were found. As such, the 95% confidence interval was used to ensure more robust conclusions.

Small changes in the mean Cobb angle and mean rib hump were found between 6 and 24 months post-surgery, but the changes in Cobb angles ranged from −6° to 3° and 7 patients had rib hump changes of 3° magnitude or more throughout this period. When the inter- and intra-vertebral rotation changes between 6 and 24 months post-surgery were compared with these clinical measures, no correlation was clearly apparent. A seemingly random spread of statistically significant inter-endplate rotation changes within and adjacent to the instrumented spine were observed and showed no correlation to the clinical measurements of rib hump or Cobb angles. Furthermore, the low frequency of rib hump recurrence was not correlated to inter-endplate rotations within these patients.

Although the incidence of significant progression of vertebral rotation after modern fusion surgery is low, when a rib hump deformity does recur, it would seem likely that several rotation deformities would recur in proximity to each other in the fused spinal levels to allow the recurrence of the hump itself; as opposed to isolated and random changes. Conversely, the entire fused spinal construct may rotate en bloc against the un-instrumented levels above and/or below thus forcing a torsional rib hump deformity to recur; which was not identified in this study.

Given the lack of a recurrence of mean rib hump deformity during the 2 years after TASF in this patient cohort, and in a previous larger TASF cohort of 106 patients followed for 2 years [18] the level of confidence in the method used for calculating inter-and intra-vertebral rotation changes seems clinically valuable. It is possible that the statistically significant changes in rotation that were observed in the current study could be due to the change in mechanics offered by the anterior fusion procedure. Given the anterolateral nature of the intervertebral fusion mass [24], stress relaxation of the spine, a gradual maturation of the fusion mass and realignment of the curve, the seemingly random pattern of inter- and intra-vertebral changes within the construct over 2 years of follow-up could conceivably be expected but not predicted.

The two patient data sets that were subsequently removed from the working pool of 34 patients because of un-correctable values of vertebral rotation (Fig. 5) would likely not have affected the results of this study given that there was no discernible pattern of inter- or intra-vertebral rotation change within or adjacent to the instrumented levels over the 2 years post-surgery and sufficient patient numbers remained after their removal.

The method for measurement of end-plate rotation used in the current study was based on a method of automatic measurement of vertebral rotation in idiopathic scoliosis on pre-operative CT scans [36]. This method was modified in the current study as automatic measurement via grey level thresholding was unable to be achieved in post-operative imaging due to the interference of ‘CT noise’ that resulted from vertebral screws and the titanium rod. Furthermore, all images were true axial images through the plane of each given end-plate (Fig. 2). Thus, observer variability was introduced during generation of true axial images and the polygonal outlining of vertebral end-plates (Fig. 3). Anterior structures only were included in the binary image analysis as posterior structures can be considered irrelevant when considering end-plate rotations; the posterior structures are also not often visualised on the true axial plane through an end-plate; and additionally posterior structures are almost always deformed in AIS patients of operable severity [42] and likely to skew results.

## Conclusions

Inter- and intra-vertebral rotation continues sporadically during the 24 months after instrumented scoliosis surgery both within the instrumented spinal levels and, less commonly, at the adjacent un-instrumented levels of the spine. No patterns for proximity of the rotational changes to the apex of the deformity or to the superior and inferior extremities of the instrumented fusion construct were found. There was no measurable en-bloc movement of the instrumented spinal levels against the adjacent un-instrumented IV levels during the 2 years following TASF for progressive AIS. This was reflected in minimal changes to the clinical measures of rib hump and Cobb angle during the 2 year post-surgery period.

The surgical construct remains relatively stable in the 24 months post-surgery as only sporadic statistically significant inter- and intra-vertebral rotation changes were observed within the instrumented levels of spine. Despite the ongoing adaptation and remodelling of the vertebrae within the fusion mass after surgery, the surgical construct maintained the gross correction of deformity achieved during surgery. Clinically, any en-bloc movement of the fused construct relative to the adjacent un-instrumented levels was not large enough to be of relevance after surgery.

## Abbreviations

AIS: Adolescent idiopathic scoliosis
CT: Computed tomography
IV: Inter-vertebral
TASF: Thoracoscopic anterior spinal fusion
